# Role of paraoxonase 1 activity and PON1 gene polymorphisms in sickle cell disease

**DOI:** 10.1038/s41598-023-34396-1

**Published:** 2023-05-03

**Authors:** Joelma Figueiredo Menezes, Magda Oliveira Seixas Carvalho, Larissa Carneiro Rocha, Felipe Miranda dos Santos, Elisângela Vitória Adorno, Cyntia Cajado de Souza, Rayra Pereira Santiago, Caroline Conceição da Guarda, Rodrigo Mota de Oliveira, Camylla Vilas Boas Figueiredo, Suéllen Pinheiro Carvalho, Sètondji Cocou Modeste Alexandre Yahouédéhou, Luciana Magalhães Fiuza, Corynne Stéphanie Ahouefa Adanho, Thassila Nogueira Pitanga, Isa Menezes Lyra, Valma Maria Lopes Nascimento, Alberto Augusto Noronha-Dutra, Marilda Souza Goncalves

**Affiliations:** 1 Laboratório de Investigação em Genética e Hematologia Translacional, Instituto Gonçalo Moniz, Fundação Oswaldo Cruz (FIOCRUZ), Salvador, Bahia Brazil; 2 Departamento de Toxicologias e Análises Clínicas, Faculdade de Farmácia, Universidade Federal da Bahia, Salvador, Bahia Brazil; 3 Fundação de Hematologia e Hemoterapia do Estado da Bahia (HEMOBA), Salvador, Bahia Brazil; 4 Hospital Universitário Professor Edgard Santos, Universidade Federal da Bahia, Salvador, Bahia Brazil; 5 University College of London, UCL, London, UK; 6 Faculdade de Medicina, Universidade Federal da Bahia, Salvador, Bahia Brazil

**Keywords:** Molecular biology, Diseases

## Abstract

Sickle cell disease (SCD) patients often exhibit a dyslipidemic sub-phenotype. Paraoxonase 1 (PON 1) is a serum glycoprotein associated with the high-density lipoproteins cholesterol (HDL-C), and variability in PON1 activity depends on the *PON1* genotypes. We investigated the influence of *PON1*c.192Q > R and *PON1*c.55L > M polymorphisms on PON1 activity and laboratory parameters and the association between PON1 activity and clinical manifestations in SCD patients. We recruited 350 individuals, including 154 SCD patients and 196 healthy volunteers, which comprised the control group. Laboratory parameters and molecular analyses were investigated from the participants' blood samples. We have found increased PON1 activity in SCD individuals compared to the control group. In addition, carriers of the variant genotype of each polymorphism presented lower PON1 activity. SCD individuals carrying the variant genotype of *PON1*c.55L > M polymorphism had lower platelet and reticulocyte counts, C-reactive protein, and aspartate aminotransferase levels; in addition to higher creatinine levels. SCD individuals carrying the variant genotype of *PON1*c.192Q > R polymorphism had lower triglyceride, VLDL-c, and indirect bilirubin levels. Furthermore, we observed an association between PON1 activity history of stroke and splenectomy. The present study confirmed the association between *PON1*c.192Q > R and *PON1*c.55L > M polymorphisms and PON1 activity, in addition to demonstrate their effects on markers of dislipidemia, hemolysis and inflammation, in SCD individuals. Moreover, data suggest PON1 activity as a potential biomarker related to stroke and splenectomy.

## Introduction

Sickle cell disease (SCD) is a genetic disorder characterized by the presence of hemoglobin S (HbS). The most frequent forms are homozygous (HbSS, sickle cell anemia) or heterozygous (HbSC, or HbSβ-thalassemia). The sickle red blood cell has complex interactions with endothelial cells, leukocytes, platelets, and plasma constituents, leading to vaso-occlusion, which is responsible for most of the clinical features and complications of the disease^1–3^. Hemoglobin S is vulnerable to slow oxidation followed by oxygen release^4–6^, resulting in chronic cycles of vascular ischemia–reperfusion and tissue injury. This direct tissue ischemia and factors such as plasma hemoglobin-induced endothelial dysfunction, free radical generation, and cytokine activation produce a characteristic chronic inflammatory state^2,7^. Previous studies suggest a dyslipidemic subphenotype in SCD that is characterized by reductions in total cholesterol, high-density lipoprotein cholesterol (HDL-C), and low-density lipoprotein cholesterol (LDL-C) and increases in VLDL-C and triglycerides. These lipid alterations were also associated with biomarkers of hemolysis and endothelial dysfunction^8,9^. Abnormal lipid homeostasis has also been suggested to change during a vaso-occlusive crisis^10–12^.

Paraoxonase-1 (PON1) is an aryldialkylphosphatase glycoprotein synthesized by the liver that circulates in the serum in association with apoA-I of the HDL-C. *PON* genes are located at chromosome 7q21–22 and contain three members: *PON1*, *PON2*, and *PON3*, and the variability of PON activity has been associated with polymorphisms in these genes^13–15^. The *PON1* gene demonstrated its crucial role in lipid metabolism, cardiovascular disease, and atherosclerosis^16^. Two polymorphisms were identified in the coding region of the *PON1* gene, the first where methionine replaces leucine at position 55 (*PON1* 55 M/L) and the second where arginine replaces glutamine at position 192 (*PON1* 192 R/Q). The frequency of *PON1* polymorphisms varies among human populations^17^. The *PON2* has two polymorphisms: the 148 G/R, where glycine replaces arginine, and the 311 C/S, where cysteine substitutes serine^17,18^.

HDL-C is a relevant cardiovascular risk factor and a fraction of total cholesterol, which consists of a group of particles originally obtained by plasma ultracentrifugation^19,20^. The main apolipoproteins of HDL-C are apoA-I and apoA-II. However, others, such as apoA-IV, apoA-V, apoC-I, apoC-II, apoC-III, apoD, and apoE, may be present^19,21^.

According to reports, PON1 possesses antioxidant and anti-inflammatory properties, as well as the ability to hydrolyze oxidized lipids in LDL-C, atherosclerotic lesions, and macrophages, preventing the initiation and progression of the atherosclerotic lesion^22–24^. The HDL has an anti-atherogenic function and some protective actions, including antioxidant protection, mediation of cholesterol efflux, inhibition of cell adhesion molecule expression and leukocyte activation, induction of nitric oxide (NO) production, regulation of blood coagulation, and platelet activity^25^. LDL-C oxidation is considered the main event involved in atherosclerosis initiation^26,27^. Oxidized LDL-C (oxLDL-C) acts as a chemotactic factor for monocytes, contributes to endothelial cell cytotoxicity, platelet activation, smooth muscle cell (SMC) migration, and proliferation, and antagonizes NO vasodilator effects^26^.

Knowing that PON1 has a crucial role in lipid metabolism and has antioxidant and anti-inflammatory properties, we aimed to investigate the influence of *PON1*c.192Q > R and *PON1*c.55L > M polymorphisms on PON1 activity and laboratory parameters and the association between PON1 activity and clinical manifestations in SCD patients.

## Methods

### Subjects and controls

The present case–control study involved 154 SCD pediatric patients. We included 101 (67.8%) individuals with sickle cell anemia (HbSS) with a mean age of 8.657 ± 0.388 and 45 (44.6%) females; 47 (31.5%) with HbSC disease, with a mean age of 10.787 ± 0.621 and 23 (48.9%) of female, and one (0.7%) four years old infant with Sβ-thalassemia. Patients attended the outpatient clinic of the Hematology and Hemotherapy Foundation of Bahia (HEMOBA). The study also included 196 healthy individuals of pediatric age that matched cases by age, gender, and African ethnic origin as a control group and were selected from individuals that attended the Clinical Laboratory College of Pharmaceutical Sciences at the Federal University of Bahia. The control group had a mean age of 9.310 (± 0.248) with 97 (49.5%) females, and all had an AA hemoglobin profile.

### Ethics

The present study was approved by the Institutional Review Board of the Gonçalo Moniz Institute, Oswaldo Cruz Foundation (Fiocruz Bahia, Brazil), and complies with the Declaration of Helsinki 1964 and its subsequent amendments. Additionally, all patient’s legal guardians provided a signed informed consent form.

### Laboratory methods

Hematological analyses were carried out using an electronic cell counter, CELL—DYN 3700 (Santa Clara, USA). Reticulocyte count was performed after blue brilliant cresyl dye staining, and hemoglobin (Hb) profile, as well as HbF levels, were investigated by high-performance liquid chromatography using an HPLC/Variant-II hemoglobin testing system (Bio-Rad, Hercules, California, USA).

Biochemical parameters were assessed from serum by spectrophotometric methods using an immunochemistry assay (A25 system, BIOSYSTEMS SA, Barcelona, Spain). C-reactive protein (CRP), anti-streptolysin-O (ASLO), and alpha 1- antitrypsin (A1AT) were assessed by immunochemistry (Immage® 800system, Beckman Coulter, Fullerton, CA). Serum ferritin was measured by immunoassay using an Access® 2 Immunoassay system X2 (Beckman Coulter, Fullerton, CA).

### PON1 activity

PON1 activity was measured by adding serum preincubated with 5 µmol/L de serine to 1 mL of tris–HCl buffer (100 mmol/L, pH 8.0) containing 2 mmol/L CaCl_2_ and 5.5 mmol/L paraoxon (O,O-diethyl-O-nitrophenyl phosphate; Sigma Chemical Co., UK)^28^. The rate of production of p-nitrophenol (nmol min-1 ml-1 serum) was determined at 25 °C, with a spectrophotometer (SpectraMax, USA) at 405 nm.

### PON1 polymorphisms

Molecular analyses were carried out on genomic DNA extracted from peripheral blood leukocytes using the Flexigen 250 kit (Qiagen, Hilden, Germany) following the manufacturer's guidelines. Specific primers were used to identify R192Q and M55L mutations to PON1.

Primers of position 192 were as follows: 5′TAT.TGT.TGC.TGT.GGG.ACC.TGA.G3′ and 5′CAC.GCT.AAA.CCC.AAA.TAC.ATC.TC3′ for 99 bp DNA; of position 55 were 5′GAA.GAG.TGA.TGT.ATA.GCC.CCA.G3′ and 5′TTT.AAT.CCA.GAG.CTA.ATG.AAA.GCC3′ for 170 bp. The PCR products were digested with AlwI to R192Q and CviAII to M55L.

### Statistical analysis

Data were expressed as mean ± standard deviation or number or percentage where appropriate. The distribution analysis of quantitative variables was investigated using the Kolmogorov–Smirnov test. An unpaired t-test was used to compare the mean values between the two groups when the distribution within these groups was normal, while Mann–Whitney was used for non-normal distribution. All analyses were performed using the Statistical Package for the Social Sciences (SPSS) v. 20.0 software (IBM, Armonk, New York, USA) and GraphPad Prism v. 6.0 (GraphPad Software, San Diego, California, USA), with *p* < 0.05 considered statistically significant.

## Results

### Laboratory characterization

Laboratory parameters, including hematologic, biochemical, and inflammatory biomarkers, of SCD individuals and the control group, are summarized in Table 1.

**Table 1:** Association of laboratory parameters in SCD individuals and controls.

Laboratory parameters	SCD (n = 154)	Control group (n = 196)	*P* value
	Mean ± SD	Mean ± SD	
Sex, % of females	70 (45.8)	97 (49.7)	–
Age, years	9.28 ± 4.05	9.32 ± 3.46	–
Hemolysis markers			
RBC, 10^12^/mL	3.23 ± 0.66	4.71 ± 0.37	**0.000**
Hemoglobin, g/dL	8.92 ± 2.00	12.72 ± 1.08	**0.000**
Hematocrit, %	27.60 ± 6.20	38.21 ± 2.87	**0.000**
MCV, fL	87.52 ± 10.81	81.17 ± 5.18	**0.000**
MCH, ρg	28.34 ± 3.75	27.02 ± 2.00	**0.000**
MCHC, g/dL	32.35 ± 0. 98	33.22 ± 1.10	**0.000**
Reticulocyte count, %	7.40 ± 4.78	0.86 ± 0.27	**0.000**
Total bilirubin, mg/dL	3.331 ± 1.782	0.506 ± 0.218	**0.000**
Direct bilirubin, mg/dL	0.777 ± 0.532	0.252 ± 0.098	**0.000**
Indirect bilirubin, mg/dL	2.553 ± 1.641	0.254 ± 0.177	**0.000**
LDH, U/L	1034.58 ± 501.95	357.51 ± 143.01	**0.000**
Leukocytes			
WBC, × 10^9^/L	13,081.81 ± 5744.82	6965.12 ± 2138.94	**0.000**
Neutrophils, × 10^9^/L	6128.33 ± 3001.36	4547.1 ± 1180.4	**0.000**
Monocytes, × 10^9^/L	811.23 ± 483.58	526.81 ± 420.18	**0.000**
Lymphocytes, × 10^9^/L	5063.20 ± 2353.85	2753.32 ± 846.87	**0.000**
Platelets			
Platelet count, × 10^3^/mL	405.539 ± 158.252	303.219 ± 67.972	**0.000**
Lipid metabolism			
Total Cholesterol, mg/dL	121.31 ± 26.46	164.41 ± 35.94	**0.000**
HDL-C, mg/dL	32.29 ± 10.03	47.03 ± 12.95	**0.000**
LDL-C, mg/dL	67.47 ± 22.37	99.32 ± 33.09	**0.001**
VLDL-C, mg/dL	22.48 ± 10.94	17.92 ± 9.79	**0.002**
Triglycerides, mg/dL	112.11 ± 54.83	89.19 ± 48.85	**0.000**
Iron metabolism			
Iron, mcg/dL	136.32 ± 126.11	74.74 ± 40.17	**0.000**
Renal profile			
Urea	17.35 ± 6.57	21.83 ± 6.06	**0.000**
Creatinine	0.44 ± 0.18	0.55 ± 0.20	**0.003**
Hepatic profile			
AST, U/L	55.72 ± 26.74	32.19 ± 10.49	**0.000**
ALT, U/L	30.45 ± 24.33	18.38 ± 7.31	**0.000**
Inflammatory biomarkers			
Ferritin, ng/dL	395.41 ± 414.65	38.08 ± 25.76	**0.000**
ASLO, UI/mL	207.78 ± 296.64	134.94 ± 137.57	**0.000**
CRP mg/L	7.62 ± 13.27	3.93 ± 15.81	**0.000**
Alpha 1-antitrypsin, mg/dL	160.83 ± 45.34	136.09 ± 39.10	**0.000**

*RBC* red blood cells, *MCV* mean cell volume, *MCH* mean corpuscular hemoglobin, *MCHC* mean corpuscular hemoglobin concentration, *RDW* red cell distribution width, *LDH* lactate dehydrogenase, *WBC* white blood cell, *HDL-C* high-density lipoprotein cholesterol, *LDL-C* low-density lipoprotein cholesterol, *VLDL-C* very low-density lipoprotein cholesterol, *AST* aspartate amino-transferase, *ALT* alanine amino-transferase, *ASLO* Anti-streptolysin O, *CRP* C-reactive protein.

Bold values indicate significance at *p* < 0.05, *p*-values obtained with Mann–Whitney.

### PON1c.192Q > R and PON1c.55L > M frequencies

Table 2 shows the frequency of *PON1c*.192Q > R and *PON1c*.55L > M variants in SCD patients and controls. The frequency of the variant *PON1c*.192R allele was 0.38 and 0.45 in SCD patients and controls, respectively. Regarding the variant *PON1c*.55 M, the allelic frequency was 0.09 and 0.15 in SCD patients and controls, respectively.

**Table 2:** Genotypic and allele frequency of *PON1* c.192Q > R and *PON1* c.55L > M among SCD patients and control group.

Polymorphism	SCD N	Genotypic frequency	Allele frequency	CG N	Genotypic frequency	Allele frequency
*PON1c.192Q* > *R*						
QQ	49	0.368	0.620	50	0.273	0.547
RR	17	0.128	0.380	33	0.180	0.453
RQ	67	0.503		100	0.547	
*PON1*c.55L > M						
LL	108	0.831	0.912	137	0.740	0.846
MM	01	0.008	0.088	09	0.049	0.154
LM	21	0.161		39	0.211	

*SCD* sickle cell disease, *CG* control group, *N* patient number.

### PON1 activity in SCD and control group

**Figure 1:**
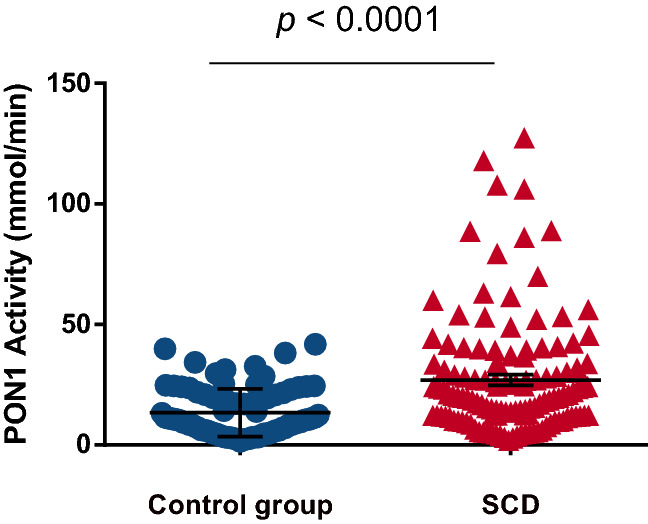
Evaluation of PON1 activity in SCD patients and control group. SCD individuals exhibited increased levels of PON1 activity (Control group n = 85 and SCD n = 124). *P*-value obtained with Mann–Whitney Test.

SCD patients showed significantly higher PON1 activity compared to the control group (Fig. 1). Analysis of PON1 activity between SCD and control group, according to the genotypes, showed interesting results. SCD patients with the heterozygote *PON1*c.192QR genotype exhibited an increase in PON1 activity compared to the control group with the same genotype (*p* = 0.027). Regarding the *PON1*c.55L > M polymorphism, SCD patients with the wild-type *PON1*c.55LL genotype presented an increase in PON1 activity in comparison with the individuals of control group carriers of the same genotype (*p* = 0.002) (Table 3).

**Table 3:** PON1 activity in different genotypes for the polymorphisms *PON1* c.192Q > R and *PON1* c.55L > M among individuals with SCD and control group.

Polymorphism	PON1 activity (mmol/min/mL)		
	SCD	Control group	*P* value
*PON1*c.192Q > R			
QQ	37.286 ± 3.854	12.458 ± 10.173	0.109
RR	11.447 ± 8.489	11.546 ± 7.561	0.144
QR	24.364 ± 8.517	14.704 ± 10.477	**0.027**
*PON1* c.55L > M			
LL	30.351 ± 27.204	14.643 ± 9.571	**0.002**
MM	14.330 ± 0.000	29.470 ± 17.409	0.252
LM	15.569 ± 7.841	6.971 ± 17.409	0.176

*SCD* sickle cell disease.

Bold values indicate significance at p < 0.05 .

**Figure 2:**
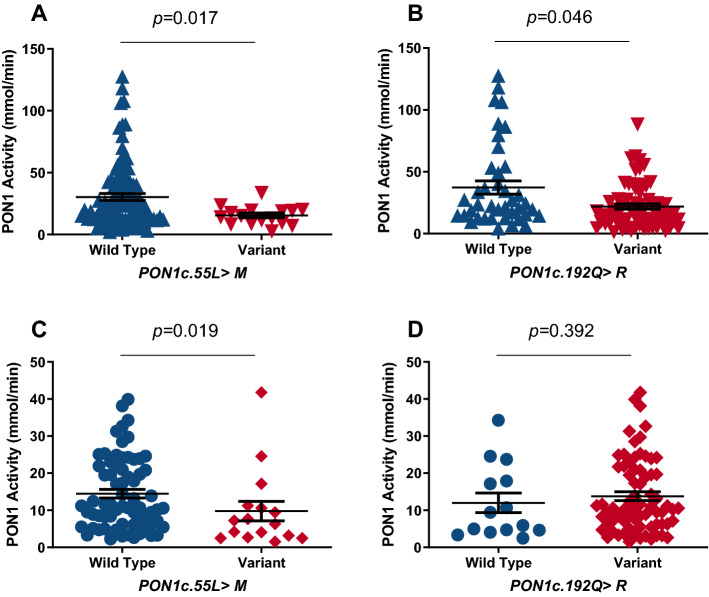
Carriers of the variant genotype of *PON1*c.55L > M and *PON1*c.192Q > R have decreased PON1 activity. (**A**) SCD individuals carrying the variant genotype of *PON1*c.55L > M presented decreased PON1 activity (wild-type = 93 and variant = 16). (**B**) SCD individuals carrying the variant genotype of *PON1*c.192Q > R exhibited decreased PON1 activity (wild-type = 40 and variant = 70). (**C**) Individuals in the control group carrying the variant genotype of *PON1*c.55L > M presented decreased PON1 activity (wild-type = 71 and variant = 16). (**D**) No statistical significance was found among carriers of the variant genotype of *PON1*c.192Q > R in the control group (wild-type = 14 and variant = 71). *PON1*c.55L > M: LL (wild type) and LM + MM (variant) genotypes. *PON1*c.192Q > R: QQ (wild-type) and (QR + RR) variant genotypes. Mann–Whitney U test was used to calculate the *P*-value.

Moreover, the variant (LM + MM) genotype of the *PON1*c.55L > M polymorphism was associated with a significant decrease in PON1 activity in both SCD and control groups (Fig. 2A,C, respectively). The variant (QR + RR) genotype of the *PON1*c.192Q > R was also associated with a significant decrease in PON1 activity in the SCD group (Fig. 2B). Noteworthy, no significant association was found between this genotype and PON1 activity in the control group (Fig. 2D).

### Association of laboratory parameters and PON1 polymorphism

**Figure 3:**
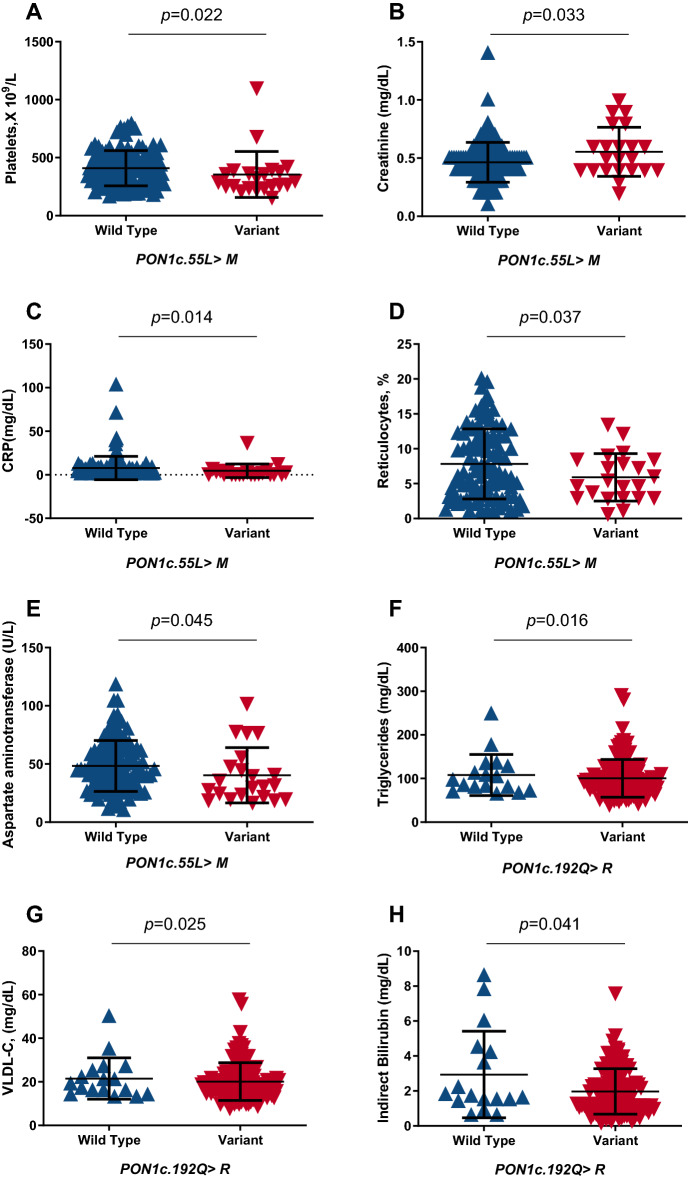
Association of laboratory parameters with different genotypes of PON1 among SCD individuals. SCD individuals with the variant genotype for *PON1*c.55L > M exhibited (**A**) decreased platelet count (wild-type = 108 and variant = 22); (**B**) increased creatinine levels (wild-type = 107 and variant = 22); (**C**) decreased CRP levels (wild-type = 108 and variant = 22); (**D**) decreased reticulocyte counts (wild-type = 96 and variant = 22); (**E**) decreased AST levels (wild-type = 108 and variant = 22). SCD individuals with the variant genotype of *PON1*c.192Q > R had (**F**) decreased triglyceride levels (wild-type = 17 and variant = 116); (**G**) decreased VLDL-c levels (wild-type = 17 and variant = 116); (**H**) decreased indirect bilirubin (wild-type = 17 and variant = 116). *PON1*c.55L > M: LL (wild type) and LM + MM (variant) genotypes. *PON1*c.192Q > R: QQ (wild-type) and (QR + RR) variant genotypes. Mann–Whitney U test was used to calculate the *P*-value.

We also investigated the association of laboratory parameters in SCD with PON1 polymorphisms. The variant (LM + MM) genotype was associated with significant decreases in platelet and reticulocyte counts, CRP, and AST levels, as well as a significant increase in creatinine levels (Fig. 3A–E). In addition, we found significant associations between the variant (QR + RR) genotype and lower triglycerides, VLDL-c, and indirect bilirubin levels (Fig. 3F–H).

### Evaluation of clinical manifestations and PON1 activity

**Figure 4:**
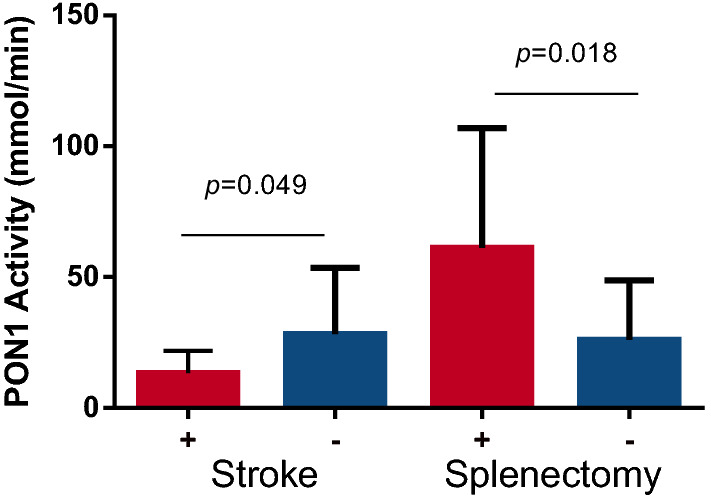
Analysis of the clinical history of individuals with SCD and PON1 activity. SCD individuals with previous stroke had decreased PON1 activity; SCD individuals with previous splenectomy exhibited increased PON1 activity. Five patients had previous stroke history, and 6 patients had undergone splenectomy. *P*-value was obtained with Mann–Whitney.

We have found that SCD individuals with previous stroke history had lower PON1 activity, while those who underwent splenectomy exhibited higher PON1 activity (Fig. 4).

## Discussion

SCD is a group of genetic diseases characterized by the presence of hemoglobin S. Under low oxygen conditions, hemoglobin S forms long polymers, which leads to red blood cell sickling. This event is central to vaso-occlusion, resulting in ischemia, pain, vaso-occlusive crises, and hemolysis^1,2,29^. SCD patients show heterogeneous clinical manifestations with serious complications related to vaso-occlusive crises, such as splenic sequestration, acute chest syndrome, ischemic stroke, priapism, and leg ulcers^1,30^. As expected, our data demonstrated statistically significant differences in hematological, biochemical, and inflammatory biomarkers when comparing SCD and the control group.

Analysis of PON1 activity demonstrated a higher level of enzymatic activity in SCD patients compared with the control group, which was corroborated by previous data^31^. A previous study showed that SCA patients had lower PON1 activity than controls^32^. The findings of Reichert et al. (2019) also suggest that SCA patients exhibit reduced PON1 activity compared to controls, although no statistical difference was found^33^. This discrepancy may be due to intrinsic characteristics related to patients and individuals included in the studies and/or the methodology used to access PON1 activity, as well as the studies’ sample size.

Allelic frequency analysis of the *PON1*c.55L > M and *PON1*c.192Q > R polymorphisms identified higher frequencies of the *PON1*c.55L (91% in SCD patients and 85% in controls) and *PON1*c.192Q (62% in SCD patients and 55% in controls) alleles. The frequencies observed in patients corroborate the findings of a previous study^33^. A study performed on Caucasians from Europe and America identified that 12%, 43%, and 45% of the individuals had QQ, QR, and RR genotypes, respectively^34^. The L allele appears to be prevalent among Japanese and Chinese (91–94%) and Caucasians (67–74%)^34^. The study of Amazonian Amerindian tribes from Brazil identified higher frequencies for L and R alleles (97% and 73%, respectively)^35^. The differences observed in frequencies may be explained by the geographical origin of the individuals included in the studies and the mixed character of the Brazilian population.

Reports suggest that *PON1* polymorphisms may influence enzymatic activity, which has been associated with conditions such as atherosclerotic coronary disease^15,35,36^. The *PON1*c.55L > M and *PON1*c.192Q > R polymorphisms investigated in the present study are known to affect the hydrolytic activity associated with lipid peroxidation. *PON1*c.55 M allele is associated with decreases in enzyme activity while *PON1*c.192R leads to an increase in its activity^36,37^. Reichert et al. also observed a significant association between higher PON activity and the variant *PON1*c.192RR genotype in patients with SCD. Contrarily, in the present study, within the SCD group, the variant *PON1*c.192R was associated with a reduction in enzyme activity. This discrepancy related to *PON1*c.192Q > R and PON activity may be explained by inherent characteristics of the individuals of the different study populations and needs to be elucidated. Moreover, SCD patients with the heterozygote *PON1*c.192QR genotype exhibited a significant increase in PON1 activity compared to the control group with the same genotype. SCD patients with the wild-type *PON1*c.55LL genotype presented a significant increase in PON1 activity compared with the individuals in the control group who carried the same genotype. Reichert et al. observed a significant decrease in PON1 activity when comparing SCA patients with heterozygote *PON1*c.55LM to control carriers of the same genotype^33^. The authors also found significant decreases in PON1 activity between SCA patients and the controls carrying the *PON1*c.192QQ and *PON1*c.192QR genotypes. Our data also demonstrated that carriers of the variant (LM + MM) genotype had lower PON1 activity than those with the wild-type LL genotype. Likewise, those with the variant (QR + RR) genotype had decreased PON1 activity compared to the wild-type QQ genotype. These findings suggest that the variant alleles may be potential risks for SCD patients.

Regarding association analyses, we observed that SCD patients with the variant (LM + MM) genotype had significant decreases in platelet and reticulocyte counts, which may be explained by the establishment of oxidative stress related to a reduction in PON1 activity presented by these patients. Indeed, it is known that the oxidative stress and decreased antioxidant capacity observed in coronary artery disease may lead to changes in platelet function^38^. The variant (LM + MM) genotype was also associated with significant decreases in CRP and AST levels, as well as a significant increase in creatinine levels, while SCD patients with the variant (QR + RR) genotype exhibited lower indirect bilirubin levels. These alterations may be due to kidney dysfunction related to impairment in PON antioxidant function.

PON is commonly related to lipid metabolism^39^. It is known that PON1 circulates in the bloodstream linked to apo A-I in HDL^27,40^, a fraction of serum lipoproteins that have an inverse relationship with total cholesterol and is responsible for the reverse transport of cholesterol circulating in the liver^25^. This mechanism is mainly involved in protection against the development of coronary disease through potentially anti-atherogenic effects. In this process, HDL inhibits monocyte chemotaxis, adherence of leukocytes, LDL oxidation, and platelet activation. The antioxidant effect of HDL on LDL was attributed to the antioxidant content of lipoproteins and the presence of PON^25^. It was shown that PON could inhibit HDL oxidation^22^. A previous report demonstrated a positive correlation between PON1 activity and HDL-C levels in SCA patients^33^. In the present study, the data did not show any significant association between the investigated polymorphisms and HDL levels. However, SCD patients with the variant (QR + RR) genotype exhibited lower VLDL-c and triglyceride levels.

The VLDL-C is a subclass of lipoprotein synthesized in the liver and responsible for transporting endogenous products (cholesterol, cholesterol esters, triglycerides, and phospholipids) in circulation, which receive the apolipoprotein E and C2 in the capillaries in contact with the lipoprotein lipase (LPL). The action of LPL is the removal of triglycerides from VLDL-c, which turns into LDL-c. The levels of VLDL have been associated with an accelerated rate of atherosclerosis and an increase in the number of diseases and metabolic states^25^.

Regarding analyses between clinical manifestations and PON1 activity in SCD patients, we observed a significant decrease in PON1 activity in those with previous stroke history or who did not undergo splenectomy. El-Ghamrawy et al. did not observe any association between PON1 activity and clinical manifestations in SCD individuals^41^. Despite the discrepancy, our data suggest the involvement of PON1 in organ injury, as it was previously reported that an impairment in antioxidant function related to PON2 deficiency could lead to vascular inflammation and abnormalities in blood coagulation^42^.

## Conclusion

Data showed that lipid metabolism is an essential component of vascular homeostasis. The PON appears to prevent the accumulation of lipoperoxide in LDL-C and thus prevent the propagation of lipid peroxidation because of the action of free radicals on oxidized LDL. In SCD patients, the presence of polymorphisms in the *PON1* gene, which alters its activity, may be associated with worsening clinical symptoms in these individuals. Further studies are needed to confirm the polymorphism of the *PON1* gene as a risk factor for patients with SCD, taking into account the diverse genetic background of the population.

## Data Availability

The datasets supporting the conclusions of this article are included in the article.

## Abbreviations

Hb: Hemoglobin
HBA: Globin-α genes
HbF: Hemoglobin fetal
HDL-C: High-density lipoprotein cholesterol
Ht: Hematocrit
LDH: Lactate dehydrogenase
LDL-C: Low-density lipoprotein cholesterol
MCH: Mean corpuscular hemoglobin
MCV: Mean cell volume
RBC: Red blood cell
SCA: Sickle cell anemia
SCD: Sickle cell disease
VLDL-C: Very low-density lipoprotein cholesterol
PON 1: Paraoxonase 1
NO: Nitric oxide
oxLDL-C: Oxidized LDL-C
SMC: Smooth muscle cell
LPL: Lipoprotein Lipase
